# Supplementary figures and images for: Effect of different long-term fertilizer managements on soil nitrogen fixing bacteria community in a double-cropping rice paddy field of southern China
Source: PLoS One. 2021 Sep 1;16(9):e0256754. doi: 10.1371/journal.pone.0256754 (PMC8409621; doi:10.1371/journal.pone.0256754)

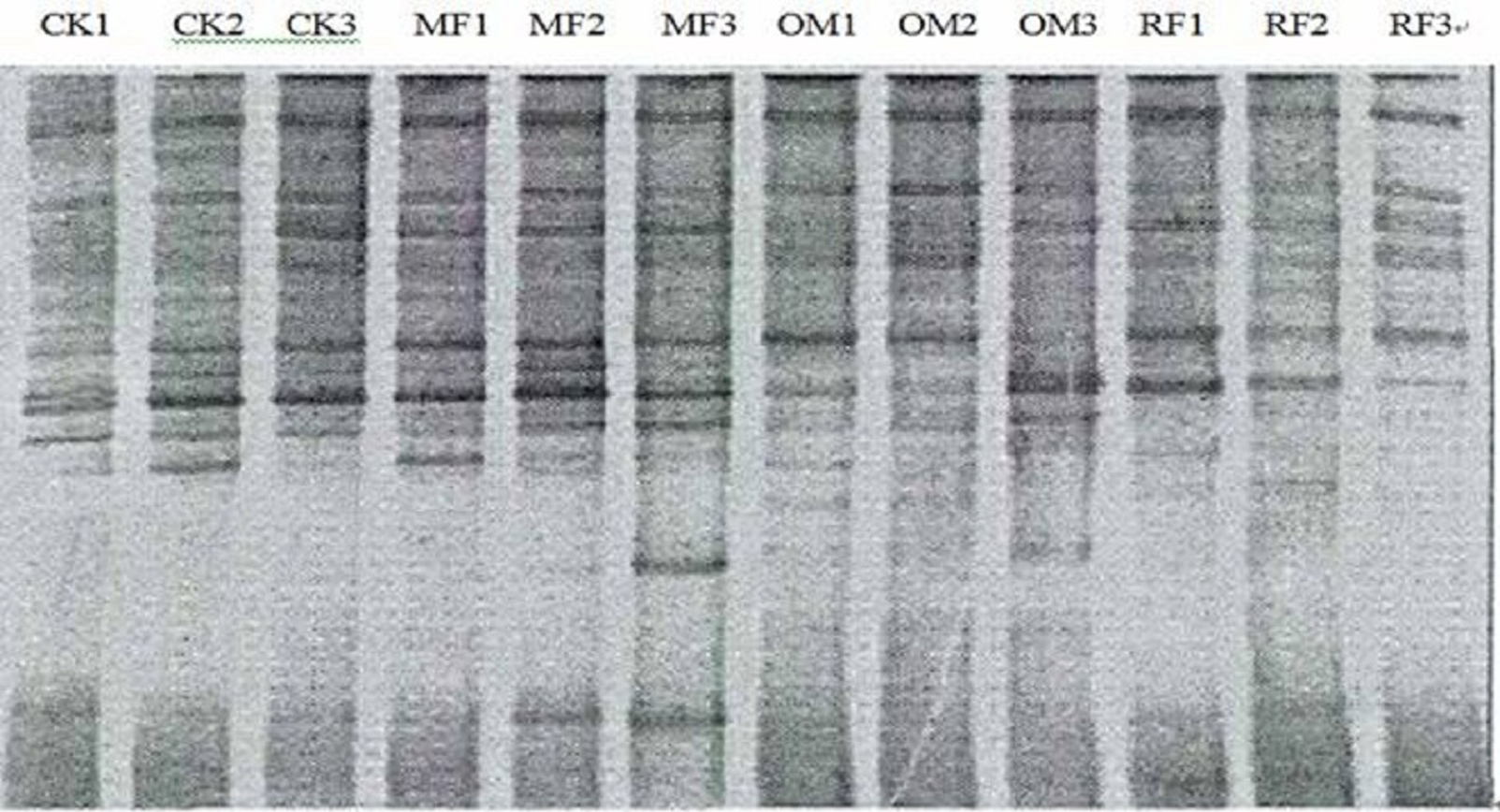

Supplement: S1 Fig — (TIF) [file pone.0256754.s001.tif]

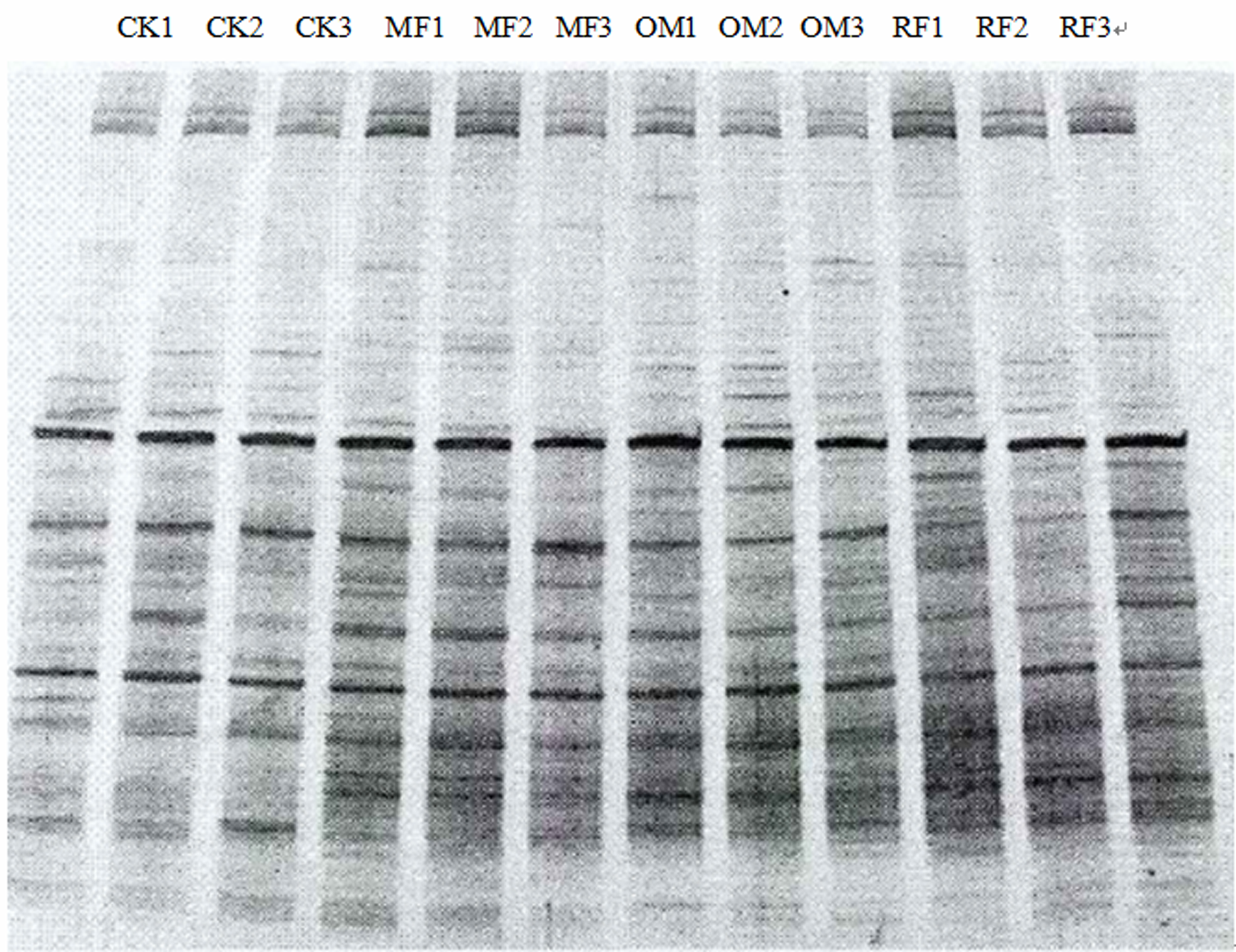

Supplement: S2 Fig — (TIF) [file pone.0256754.s002.tif]
