# Supplementary material for: Effect of different long-term fertilizer managements on soil nitrogen fixing bacteria community in a double-cropping rice paddy field of southern China
Source: PLoS One. 2021 Sep 1;16(9):e0256754. doi: 10.1371/journal.pone.0256754 (PMC8409621; doi:10.1371/journal.pone.0256754)
Supplement: Attachment — Submitted filename: PONE-D-20-36146_reviewer_Revised.pdf [file pone.0256754.s003.pdf]

PLOS ONE

Effect of different long-term fertilizer management on nitrogen fixing bacteria community in a double-cropping paddy field of southern China

--Manuscript Draft--

**Manuscript Number:**	PONE-D-20-36146
**Article Type:**	Research Article
**Full Title:**	Effect of different long-term fertilizer management on nitrogen fixing bacteria community in a double-cropping paddy field of southern China
**Short Title:**	nitrogen fixing bacteria community
**Corresponding Author:**	Haiming Tang<br>Hunan Soil and Fertilizer Institute<br>Changsha, CHINA
**Keywords:**	rice; fertilizer treatment; N fixing bacteria; nifH gene; paddy field
**Abstract:**	While nitrogen (N) fixation by soil microorganisms is an important N cycling processes, but there is still limited information on how the soil microbes that drive this processes respond to soil physical and chemical properties changes under double-cropping rice (Oryza sativa L.) paddy field in southern China. Therefore, the 34-year long-term fertilizer regime on nitrogen fixing bacteria community under double-cropping rice field in southern China were studied by using PCR-DGGE method in the present paper. The field experiment were including four different fertilizer treatments: chemical fertilizer alone (MF), rice straw residue and chemical fertilizer (RF), 30% organic manure and 70% chemical fertilizer (OM), and without fertilizer input as control (CK). The results showed that the diversity index of cbbLR gene and nifH gene were increased with RF and OM treatments, compared with CK treatment. Compared with CK treatment, the abundance of cbbLR gene were increased by 6.70, 12.19 and 23.94×107 copies g-1 with MF, RF and OM treatments, respectively. Meanwhile, the abundance of nifH gene were increased by 5.40, 8.82 and 23.90×109 copies g-1 with MF, RF and OM treatments, compared with CK treatment, respectively. The results also indicated that soil autotrophic azotobacter and nitrogenase activities with RF and OM treatments were significantly higher ($p<0.05$) than that of CK treatments. There is an obvious difference in characteristic of N fixing bacteria community between application of inorganic fertilizers and organic manure treatments. In summary, the results indicated that abundances of N fixing bacteria community in the double-cropping rice paddy soil were increased by application of crop residue and organic manure practice.
**Order of Authors:**	Haiming Tang
	Chao Li
	Lihong Shi
	Xiaoping Xiao
	Kaikai Cheng
	Li Wen
	Weiyan Li
**Additional Information:**	
**Question**	**Response**
**Financial Disclosure**<br><br>Enter a financial disclosure statement that describes the sources of funding for the work included in this submission. Review the submission guidelines for detailed requirements. View published research	The author(s) received no specific funding for this work.

articles from *PLOS ONE* for specific examples.<br><br>This statement is required for submission and **will appear in the published article** if the submission is accepted. Please make sure it is accurate.<br><br>**Unfunded studies**<br>Enter: *The author(s) received no specific funding for this work.*<br><br>**Funded studies**<br>Enter a statement with the following details:<br>• Initials of the authors who received each award<br>• Grant numbers awarded to each author<br>• The full name of each funder<br>• URL of each funder website<br>• Did the sponsors or funders play any role in the study design, data collection and analysis, decision to publish, or preparation of the manuscript?<br>• **NO** - Include this sentence at the end of your statement: *The funders had no role in study design, data collection and analysis, decision to publish, or preparation of the manuscript.*<br>• **YES** - Specify the role(s) played.<br><br>* typeset	
**Competing Interests**<br><br>Use the instructions below to enter a competing interest statement for this submission. On behalf of all authors, disclose any competing interests that could be perceived to bias this work—acknowledging all financial support and any other relevant financial or non-financial competing interests.<br><br>This statement **will appear in the published article** if the submission is accepted. Please make sure it is accurate. View published research articles from *PLOS ONE* for specific examples.	The authors have declared that no competing interests exist.

**NO authors have competing interests**<br><br>Enter: *The authors have declared that no competing interests exist.*<br><br>**Authors with competing interests**<br><br>Enter competing interest details beginning with this statement:<br><br>*I have read the journal's policy and the authors of this manuscript have the following competing interests: [insert competing interests here]*<br><br>* typeset	
**Ethics Statement**<br><br>Enter an ethics statement for this submission. This statement is required if the study involved:<br><br>• Human participants<br>• Human specimens or tissue<br>• Vertebrate animals or cephalopods<br>• Vertebrate embryos or tissues<br>• Field research<br><br>Write "N/A" if the submission does not require an ethics statement.<br><br>General guidance is provided below. Consult the submission guidelines for detailed instructions. **Make sure that all information entered here is included in the Methods section of the manuscript.**	N/A

**Format for specific study types**<br><br>**Human Subject Research (involving human participants and/or tissue)**<br>• Give the name of the institutional review board or ethics committee that approved the study<br>• Include the approval number and/or a statement indicating approval of this research<br>• Indicate the form of consent obtained (written/oral) or the reason that consent was not obtained (e.g. the data were analyzed anonymously)<br><br>**Animal Research (involving vertebrate animals, embryos or tissues)**<br>• Provide the name of the Institutional Animal Care and Use Committee (IACUC) or other relevant ethics board that reviewed the study protocol, and indicate whether they approved this research or granted a formal waiver of ethical approval<br>• Include an approval number if one was obtained<br>• If the study involved *non-human primates*, add *additional details* about animal welfare and steps taken to ameliorate suffering<br>• If anesthesia, euthanasia, or any kind of animal sacrifice is part of the study, include briefly which substances and/or methods were applied<br><br>**Field Research**<br><br>Include the following details if this study involves the collection of plant, animal, or other materials from a natural setting:<br>• Field permit number<br>• Name of the institution or relevant body that granted permission	
**Data Availability**<br><br>Authors are required to make all data underlying the findings described fully available, without restriction, and from the time of publication. PLOS allows rare exceptions to address legal and ethical concerns. See the PLOS Data Policy and FAQ for detailed information.	Yes - all data are fully available without restriction

Powered by Editorial Manager® and ProduXion Manager® from Aries Systems Corporation

A Data Availability Statement describing where the data can be found is required at submission. Your answers to this question constitute the Data Availability Statement and **will be published in the article**, if accepted.<br><br>**Important:** Stating 'data available on request from the author' is not sufficient. If your data are only available upon request, select 'No' for the first question and explain your exceptional situation in the text box.<br><br>Do the authors confirm that all data underlying the findings described in their manuscript are fully available without restriction?	
**Describe where the data may be found in full sentences. If you are copying our sample text, replace any instances of XXX with the appropriate details.**<br><br>• If the data are **held or will be held in a public repository**, include URLs, accession numbers or DOIs. If this information will only be available after acceptance, indicate this by ticking the box below. For example: *All XXX files are available from the XXX database (accession number(s) XXX, XXX.).*<br>• If the data are all contained **within the manuscript and/or Supporting Information files**, enter the following: *All relevant data are within the manuscript and its Supporting Information files.*<br>• If neither of these applies but you are able to provide **details of access elsewhere**, with or without limitations, please do so. For example:<br><br>*Data cannot be shared publicly because of [XXX]. Data are available from the XXX Institutional Data Access / Ethics Committee (contact via XXX) for researchers who meet the criteria for access to confidential data.*<br><br>*The data underlying the results presented in the study are available from (include the name of the third party*	All relevant data are within the manuscript and its Supporting Information files.

and contact information or URL).<br>• This text is appropriate if the data are owned by a third party and authors do not have permission to share the data.<br><br>* typeset	
Additional data availability information:	

Manuscript Click here to access/download;Manuscript;renamed_42399.doc

Title page

Title

Effect of different long-term fertilizer management on nitrogen fixing bacteria community in a double-cropping paddy field of southern China

Author names

Haiming Tang, Chao Li, Lihong Shi, Xiaoping Xiao, Kaikai Cheng, Li Wen, Weiyan Li

Affiliations

Hunan Soil and Fertilizer Institute, Changsha 410125, China

*** Corresponding author**

Haiming Tang

Tel: +86 731 84696102; Fax: +86 731 84691581; E-Mail address: tanghaiming66@163.com.

Effect of different long-term fertilizer management on nitrogen fixing bacteria community in a double-cropping paddy field of southern China

Abstract: ~~While~~ nitrogen (N) fixation by soil microorganisms is an important N cycling processes, but there is still limited information on how the soil microbes that drive this processes respond to soil physical and chemical properties changes under double-cropping rice (*Oryza sativa* L.) paddy field in southern China. Therefore, ~~the~~ 34-year long-term fertilizer regime on nitrogen fixing bacteria community under double-cropping rice field in southern China ~~were~~ studied by using PCR-DGGE method in the present paper. The field experiment ~~were including~~ four different fertilizer treatments: chemical fertilizer alone (MF), rice straw residue and chemical fertilizer (RF), 30% organic manure and 70% chemical fertilizer (OM), and ~~without fertilizer input as~~ control (CK). The results showed that the diversity index of *cbbL*R gene and *nifH* gene were increased with RF and OM treatments, compared with CK treatment. Compared with CK treatment, the abundance of *cbbL*R gene were increased by 6.70, 12.19 and 23.94×10^7 copies g^{-1} with MF, RF and OM treatments, respectively. Meanwhile, the abundance of *nifH* gene were increased by 5.40, 8.82 and 23.90×10^9 copies g^{-1} with MF, RF and OM treatments, compared with CK treatment, respectively. The results also indicated that soil autotrophic azotobacter and nitrogenase activities with RF and OM treatments were significantly higher ($p<0.05$) than that of CK treatments. There is an ~~obvious~~ difference in characteristic of N fixing bacteria community between application of inorganic fertilizers and organic manure treatments. In summary, the results indicated that abundances of N fixing bacteria community in the double-cropping rice paddy soil were increased by application of crop residue and organic manure practice.

Key words: rice; fertilizer treatment; N fixing bacteria; *nifH* gene; paddy field

Introduction

Biological nitrogen (N) fixation, the conversion of nitrogen gas (N_2) into ammonia mediated by bacteria, is considered an important way to maintain a reliable N-supply for rice growth in paddy soil [1]. The N accumulation per year via biological N_2-fixation in paddy field was 32 kg hm^{-2} higher than that ~~of~~ in upland soil [2]. This is attributed to the stimulation of N_2-fixing bacteria (*cbbL*R) and nitrogenase (*nifH*) activity under anaerobic flooded condition [3-4]. Previous

studies found that bacteria with N_2-fixation capacity were distributed through diverse prokaryotic taxa including but not limited to *Proteobacteria* (α-, β-, γ- and δ-proteobacteria), phototrophic Cyanobacteria and Clostridia [1, 5-6]. The succession of N_2-fixing bacteria throughout the entire period of paddy field remains largely not clear, understanding the succession of the N_2-fixing bacterial community during the paddy field and enhancing biological N_2-fixation will be conducive to improve rice production with minimal N-fertilizer application [7].

In recent years, the rapid development of 16S rRNA gene sequencing technology and functional prediction has provided insight into community structure and function of bacteria in soil [8]. Molecular approaches have been developed and successfully applied to describe diazotroph communities, such as quantitative polymerase chain reaction (qPCR) and cloning [9], denaturing gradient gel electrophoresis (DGGE) [10], PCR-restriction fragment length polymorphism (RFLP), and fluorescently labelled terminal (FLP)-RFLP [11-12]. These approaches provide a more complete picture of the diazptrophic communities in various environments than do cultured-based approaches, such as soil, continental margin sediments, and the rhizosphere of native wetland species [13]. These studies found that nitrogen-fixing bacteria occur predominantly in the upper soil layer (5 cm depth) and are estimated to make up about 5% of the total bacterial population, as well as showing that environmental factors affected the activity and community of nitrogen-fixing bacteria, such as soil biogeochemical properties [12].

Rice (*Oryza sativa* L.) is one of the main crops in Asia, and double-cropping rice system (early rice and late rice) is the main land use in southern China [14]. It is benefit practices for maintaining or improving the paddy soil quality and fertility by application of fertilizer management (organic fertilizer, inorganic fertilizer). And the different fertilizer management may profound effects on soil physical and chemical characteristics such as pH, soil bulk density, SOC content [15], which in return affect nitrogen fixation and soil microbiological properties. Therefore, the 34-year long-term field experiment with different fertilizer treatments were conducted in a double-cropping rice system in the southern China. Hence, the objective of this study was: (1) to investigate the diversity of nitrogen-fixing bacteria in rice non-rhizosphere soil under different long-term fertilization conditions; (2) to analysis molecular diversity of the *cbbL*R and *nifH* gene and its phylogenetic with different fertilizer practices in a double-cropping rice system by using PCR-DGGE technology.

Materials and methods

Sites and cropping system

The experiment ~~was beginning in 1986. It~~ was located in NingXiang County (28°07′ N, 112°18′ E) of Hunan Province, China. The climatic conditions of the experiment field, the ~~surface~~ soil physical and chemical properties (0–20 cm) ~~beginning of~~ this experiment and crop rotation systems ~~as~~ described by Tang *et al*. (2018) [16].

Experimental design

The experiment ~~including~~ four fertilizer treatments: chemical fertilizer alone (MF), rice straw residue and chemical fertilizer (RF), ~~and~~ 30% organic manure and 70% chemical fertilizer (OM), ~~without fertilizer input as~~ control (CK). A randomized ~~block design was adopted in the plots~~, with three replications of each treatment~~. And~~ each plot size was 66.7 m^2 (10 × 6.67 m). The experiment ensured ~~that~~ the same amount of N, phosphorus pentoxide (P_2O_5), potassium oxide (K_2O) for all fertilizer treatments during early and late rice growing season~~, respectively~~. ~~Details~~ information about fertilizer management and ~~filed arrangement as~~ described by Tang *et al*. (2018) [16].

Soil sampling and sample preparation

Soil samples were collected at tillering stage of late rice in 2019. The soil samples were taken ~~from adjacent~~ to the rice plants (i.e. non-rhizosphere soil) ~~were sampled~~ at ~~depth 0-20 cm~~. Correspondingly, one composite soil consisting of twenty cores was taken from each plot~~. Thus~~, three composite samples of soils with each fertilizer treatment were collected at sampling time. The fresh samples were placed immediately in ice box and transported to the laboratory. The mixed samples were split into two parts: one part was stored at 4℃ for subsequent soil azotobacter and enzyme activity assays, and the other was stored at -80℃ for use in molecular experiments.

Soil laboratory analysis

Soil autotrophic azotobacter and nitrogenase activities

Soil autotrophic azotobacter was determined described by Li et al. (2008) [17]. The number of soil autotrophic azotobacter was determined by using plate count method, and the medium was Ashby medium, which was expressed by the number of colony per gram of fresh soil (cfu g^{-1}).

The soil nitrogenase activity was determined using the acetylene (C_2H_2) reduction technique

described by Schwinghamer et al. (1980) [18], but with some modifications. The fresh compost sample (7 g) was incubated in a 100 mL sterile flask with a rubber stopper. The compost sample was then amended with a solution containing glucose to obtain 1 mg C/g compost. Next, 10% of the air in the flask was replaced by acetylene gas and the flask was incubated in the dark at 28℃ for 48 h. The ethylene (C_2H_4) generated was determined by gas chromatography using a flame ionization detector (Trace GC UItra, Thermo-Fisher, USA). The nitrogenase activity was expressed as 1 nmol C_2H_4 per hour per gram of dry sample (C_2H_4 nmol/(g·h)).

Genomic DNA extraction and qPCR detection of the *cbbL*R gene and *nifH* gene

Before extracting the DNA, all of the compost samples were freeze-dried in a freeze dryer (Songyuan, Beijing, China) to ensure that the water content was at the same low level. The freeze-dried samples were then crushed and sieved through 1-mm pore filters using an ultra-centrifugal mill (ZM200, Retsch, Germany). DNA was extracted from the total microbial community using 0.5 g of the freeze-dried samples with a FastDNA Spin Kit for soil (MP Biomedicals, LLC, Illkirch, France), according to the manufacturer’s instructions. The concentration and quality of the DNA were determined using an Epoch Multi-Volume Spectrophotometer System (BioTek, USA). The extracted DNA was stored at -20℃ for subsequent use.

The copy numbers of the *nifH* gene in nitrogen-fixing bacteria were determined by qPCR, which was conducted in triplicate with an iCycler IQ5 Thermocycler (Bio-Rad, USA) using the primer sets: PolF (5′-TGCGAYCCSAARGCBGACTC-3′) and PolR (5′-ATSGCCATCA TYTCRCCGGA-3′) [19]. Each reaction system comprised a 20 μL volume containing 10 μL of 2 × UltraSYBR Mixture (Cwbiotech, Beijing, China), 0.4 μL (10 μM) of each primer, 2 μL of DNA template, and the final volume was adjusted with sterile water. The qPCR reactions were conducted with an initial denaturation step at 95℃ for 10 min, followed by 40 cycles at 95℃ for 10 s, 60℃ for 30 s, and 72℃ for 32 s. The data were retrieved at 72℃. The qPCR reaction was repeated in triplicate.

The copy numbers of the *cbbL*R gene in nitrogen-fixing bacteria were determined by qPCR, which was conducted in triplicate with an iCycler IQ5 Thermocycler (Bio-Rad, USA) using the primer sets: *cbbL*R (5′-AAG GAY GAC GAG AAC ATC-3′) and *cbbL*RintR (5′-TGC AGS ATC ATG TCR TT-3′). The PCR reaction system of *cbbL*R gene amplification is the same as that of

nifH gene. The qPCR reactions were conducted with an initial denaturation step at 95℃ for 15 min, followed by 40 cycles at 91℃ for 1 min, 55℃ for 1 min, and 72℃ for 2 min. The data were retrieved at 68℃ for 10 min. The correct length of PCR product was detected in 1.5% agarose gel electrophoretic.

The PCR products amplified from soil were used to obtain a standard curve for the *nifH* gene product. The standard curve for qPCR was produced using tenfold serial dilution of linearized plasmids containing the cloned *nifH* genes. The range of template copies used to generate the standard curve was 1.34×10^5 to 1.34×10^9 copies of template. A melting curve was obtained at the end of the reaction to verify the specificity of the amplicon. The standard curve indicated a PCR amplification efficiency of about 88.5% and linearity of 0.99. The PCR products amplified from soil were used to obtain a standard curve for the *cbbL*R gene product. The standard curve for qPCR was produced using tenfold serial dilution of linearized plasmids containing the cloned *cbbL*R genes. The range of template copies used to generate the standard curve was 3.10×10^5 to 3.10×10^9 copies of template. A melting curve was obtained at the end of the reaction to verify the specificity of the amplicon. The standard curve indicated a PCR amplification efficiency of about 98.5% and linearity of 0.99.

PCR and DGGE analysis

The primer sets used for PCR amplification were the same as those employed for qPCR. A GC clamp was attached to the forward primer (CGCCCGG GGCGCGCC CCGGGCGGGGCGGG GGCACGGGGGG) to prevent complete separation of the DNA strands during DGGE [20]. Each PCR reaction was performed in a 20 μL reaction mixture containing 1 μL of DNA template, 10 μL of 2 × Power Taq PCR MasterMix (Cwbiotech, Beijing, China), 0.4 μL (10 μM) of each primer, and the final volume was adjusted to 20 μL with sterile water. PCR amplification was performed with a MyCycler thermal cycle (Bio-Rad, Hercules, CA, USA) using the following cycling conditions: initial denaturation at 94℃ for 5 min, 40 cycles of denaturation at 94℃ for 1 min, annealing at 58℃ for 1.5 min, and extension at 72℃ for 1.5 min, followed by a final extension at 72℃ for 10 min and cooling to 4℃. The PCR product size was determined by electrophoresis on 1% agarose gel.

DGGE was performed using a Dcode™ Universal Detection System instrument according to the manufacturer's instructions (Bio-Rad, USA). The 20 μL PCR products were loaded onto 8%

polyacrylamide gel with a denaturing gradient of 30–60%. Electrophoresis was conducted in 1× TAE buffer at 60℃ for 12 h at a constant voltage of 80 V (DcodeTM Universal Detection System, Bio-Rad, USA). After electrophoresis, the gels were stained with 1: 10000 DuRed (Sigma, USA) for 30 min in the dark and then photographed under UV light using the Gel Doc XR System (Bio-Rad, Hercules, CA, USA). The results of the PCR-DGGE profiles of soil microbial *cbbL*R gene and *nifH* gene were shown in Fig. S1 and Fig. S2.

Cloning and sequencing

After DGGE, the prominent bands were excised from the gel. The bands were triturated in TE buffer (30 μL) and stored overnight at 4℃. The supernatant was employed as the template for PCR to sequence DNA bands using primer sets without the GC-clamp. Suitable PCR products were submitted to OE Biotech Company (Shanghai, China) for sequencing. The sequences were then assembled and compared using BLAST via the National Center for Biotechnology Information (NCBI) (http://www.ncbi.nlm.nih.gov). Sequence analysis and operational taxonomic units (OTUs) identification were according to the methods of Fagen et al. (2012) [21]. A neighbor-joining tree established using MEGA4 software with the Kimura two-parameter method was generated to create phylogenetic reconstructions. A bootstrap consensus tree inferred from 1000 replicates was obtained to represent the evolutionary history of the taxa analyzed.

Statistical analysis

DGGE images were analyzed by using Quantity One (version 4.6.2, Bio-Rad, USA). The similarities of the community fingerprints were calculated by using the unweighted pair group method with arithmetic mean to analyze the hierarchical clusters. The diversity of nitrogen-fixing bacteria was determined with the Diversity index, Richness index, and Evenness index [22].

The statistical analyses of this manuscript were conducted by using SAS 9.3 software package [23]. The ~~data of~~ different fertilizer treatments means ~~in this manuscript~~ were compared by using one-way analysis of variance (ANOVA) following standard procedures at the p<0.05 probability level. The results were expressed as means and standard errors.

Results

Soil autotrophic azotobacter and nitrogenase activities

The number of soil autotrophic azotobacter ranged from 6.25 to 17.34 $\times 10^5$ cfu/g, in the

different fertilizer treatments, the number of soil autotrophic azotobacter in the MF, RF and OM treatments were 1.88, 2.77 and 2.27 times that of CK treatment, respectively. The results showed that number of soil autotrophic azotobacter with RF treatment were significantly higher (p<0.05) than that of MF, OM, CK treatments. And the number of soil autotrophic azotobacter with OM, MF treatments were significantly higher (p<0.05) than that of CK treatment (Fig. 1, a).

The soil nitrogenase activities ranged from 2.54 to 8.13 C_2H_4 nmol/(g·h), in the different fertilizer treatments, the soil nitrogenase activities in the MF, RF and OM treatments were 1.74, 3.09 and 3.20 times that of CK treatment, respectively. The results showed that soil nitrogenase activities with RF, OM treatments were significantly higher (p<0.05) than that of MF, CK treatments. And the soil nitrogenase activities with MF treatment were significantly higher (p<0.05) than that of CK treatment (Fig. 1, b).

Diversities of *cbbL*R and *nifH* gene

The diversities of *cbbL*R gene and *nifH* gene as affected by different long-term fertilizer treatments were showed in Table 1. The diversity index of *cbbL*R gene with MF treatment were significantly higher (p<0.05) than that of CK treatment. Compared with CK treatment, the diversity index of *cbbL*R gene with MF treatment were increased 11.38%. And the richness of *cbbL*R gene with MF, RF and OM treatments were significantly higher (p<0.05) than that of CK treatment. Meanwhile, there is not significantly difference (p>0.05) in evenness of *cbbL*R gene among MF, RF, OM and CK treatments.

The diversity index of *nifH* gene with MF treatment were significantly higher (p<0.05) than that of CK treatment. Compared with CK treatment, the diversity index of *nifH* gene with MF treatment were increased 11.22%. And the richness of *nifH* gene with MF, RF and OM treatments were significantly higher (p<0.05) than that of CK treatment. Meanwhile, there is not significantly difference (p>0.05) in evenness of *nifH* gene among MF, RF, OM and CK treatments. That is, the diversity index of *cbbL*R gene and *nifH* gene were increased under application fertilizer condition.

Table 1 Diversities of *cbbL*R and *nifH* gene as affected by different long-term fertilizer treatments

Treatments	*cbbL*R			*nifH*		
	Diversity index	Richness index	Evenness index	Diversity index	Richness index	Evenness index
CK	3.25±0.10b	2.58±0.09b	2.26±0.07a	3.12±0.09b	2.51±0.06b	2.25±0.07a
MF	3.62±0.09a	3.22±0.07a	2.22±0.06a	3.47±0.10a	2.76±0.08a	2.24±0.06a

RF	3.47±0.10ab	3.17±0.09a	2.29±0.07a	3.43±0.10ab	2.70±0.08a	2.28±0.07a
OM	3.55±0.10ab	3.11±0.09a	2.31±0.07a	3.35±0.10ab	2.65±0.07a	2.32±0.07a

MF: chemical fertilizer alone; RF: rice straw residue and chemical fertilizer; OM: 30% organic manure and 70% chemical fertilizer; CK: without fertilizer input as control.

Values are presented as mean ± standard error.

Different letters in the same column indicate significant difference at $p<0.05$.

~~The same as below.~~

Abundance of *cbbL*R and *nifH* gene

There were ~~obvious~~ effects of different long-term fertilizer treatments on abundance of *cbbL*R gene and *nifH* gene (Table 2). The abundance of *cbbL*R gene and *nifH* gene with OM treatment ~~were~~ significantly higher ($p<0.05$) than that of MF, RF CK treatments. And the abundance of *cbbL*R gene and *nifH* gene with MF, RF treatments ~~were~~ significantly higher ($p<0.05$) than that of CK treatment, and the order of abundance of *cbbL*R gene and *nifH* gene with different fertilizer treatments was showed OM>RF>MF>CK.

The abundance of *cbbL*R gene ranged from 1.42 to 25.36 $\times10^7$ copies/g, in the different fertilizer treatments, the abundance of *cbbL*R gene in the MF, RF and OM treatments ~~were~~ 5.72, 9.58 and 17.86 times that of CK treatment, respectively. The abundance of *nifH* gene ranged from 2.45 to 26.35 $\times10^9$ copies/g, in the different fertilizer treatments, the abundance of *nifH* gene in the MF, RF and OM treatments ~~were~~ 3.20, 4.60 and 10.76 times that of CK treatment, respectively.

Table 2 Abundance of *cbbL*R and *nifH* gene as affected by different long-term fertilizer treatments

Gene	Treatments			
	MF	RF	OM	CK
*cbbL*R ($\times10^7$ copies/g)	8.12±0.43c	13.61±0.39b	25.36±0.23a	1.42±0.04d
nifH ($\times10^9$ copies/g)	7.85±0.49c	11.27±0.33b	26.35±0.22a	2.45±0.05d

~~Different letters in the same line indicate significant difference at $p<0.05$.~~

Cluster analysis of *cbbL*R and *nifH* gene

The community structures of *cbbL*R gene and *nifH* gene in soils were characterized by cluster analysis (Fig. 2 a, b). The results indicated that inorganic fertilizer and organic manure had a variety of effects on the soil *cbbL*R gene, which revealed that RF, CK treatments were different from MF and OM treatments (Fig. 2 a). A similar variation in the *nifH* gene structure was detected

among different fertilizer regime (Fig. 2 b). These findings indicated that the fertilizer regime might be a major factor affecting the soil *cbbL*R gene and *nifH* gene structure. The results revealed three major clusters with different fertilizer regime, MF and OM treatment can well aggregate into one cluster, which showed that *cbbL*R gene or *nifH* gene have relatively high similarity in community structure under the two fertilizer treatments.

Community structure of *cbbL*R and *nifH* gene

To identify the ~~different of fertilizer regime on~~ *cbbL*R gene and *nifH* gene community composition, we conducted the neighbor-joining phylogenetic analysis (Fig. 3, Fig. 4) based on the taxonomic affiliations of the sequences obtained in this study, using all detected OTUs. The results indicated that the main dominant flora of *cbbL*R gene belongs to the *Betaproteobacteria*, *Pseudoacidovorax*, *Azospira* and *Ideonella*. Interestingly, the results showed that ~~the most genus~~ were affiliated with the cluster 1 lineage, and some ~~genus~~ were affiliated with the cluster 2 lineage, cluster 3 lineage and cluster 4 lineage in the soil samples (Fig. 3). In the first cluster, Band 9, Band 10, Band 11, Band 13 were similar to nitrogen fixing bacteria HM565436.3, Band 5 was similar to bacteria HQ335728.2, Band 14 was similar to *Ideolla decloratans* strain EU542647.1. In the second cluster, Band 4, Band 6 and Band 7 were clustered with *Pseudoacidovorax*. And Band 1, Band 5, Band 8 and *Azospira* were clustered in the third cluster, and Band 12, Band 15 were different from other sequences.

According to the sequence of *nifH* gene and the most similar sequence in GenBank, the phylogenetic analysis showed that ~~the most genus~~ belonged to the cluster 1, Band 2, 3 and Band 6 belong to cluster 2, while Band 1 and Band 4-5 belong to cluster 3. In the database of GenBank, the gene sequences of cultivated microorganisms similar to Band 3 and Band 4 can be retrieved, the gene sequences of *myzf* of the similar cultivated microorganisms can not be retrieved from the other sequences, and only some of the sequences of uncultured microorganisms are similar to them.

Discussion

Effects of fertilizer regime on soil autotrophic azotobacter and nitrogenase activities

~~In the present study, this result~~ indicated that number of soil autotrophic azotobacter were significantly enhanced under long-term application of organic manure and crop residue condition,

which were agree with Yuan et al. (2011) [24], the reason maybe that the number of soil autotrophic azotobacter were stimulated under application of fertilizer condition, which provide energy substrates for soil autotrophic azotobacter growth. In the different fertilizer treatments, number of soil autotrophic azotobacter with OM and RF treatments were higher than that of the other fertilizer treatments, this phenomenon were suggestion that SOC content was important factor for autotrophic azotobacter growth [15]. On the other hand, the total input of organic carbon of crop residue carbon and organic fertilizer source carbon were significantly increased, and the components of organic carbon also changed [25]. In the present study, the results indicated that number of soil autotrophic azotobacter with MF treatment were lower than that of OM and RF treatments, which suggested that autotrophic azotobacter in paddy field were restricted under long-term application of chemical fertilizer condition.

The soil nitrogenase activity is an important indicator for estimating the capacity for biological nitrogen fixation [8]. In the present study, this result indicated that soil nitrogenase activities were significantly enhanced under long-term application of organic manure and crop residue condition, higher residual organic matter in OM, RF and MF soil than without fertilizer input soil might result in higher the nitrogenase activity in OM, RF and MF soil than CK soil [26]. The reason maybe that nitrogen fixing activity of soil nitrogen fixing microorganism were stimulated under application of fertilizer condition, which provide energy substrates for soil nitrogenase growth. Meanwhile, in the different fertilizer treatments, this study indicated that soil nitrogenase activity with OM treatment were higher than that of the other treatments, this phenomenon were suggestion that SOC content was an important factor promote the growth of nitrogenase. The nitrogenase activity with RF treatment were lower than that of OM treatment, the reason maybe that RF treatment may stimulate the nitrogen fixation activity of soil microorganism, but the inhibition of N content on nitrogenase activity is higher than that of crop residue effects, so the activity of nitrogenase were decreased. These again confirmed the succession of N_2-fixing bacteria along with the ongoing consumption of organic manure in paddy soil. However, the soil nitrogenase activity were inhibited under application of chemical fertilizer condition compared with application of organic manure and crop residue management, which were suggestion that nitrogenase activities were decrease with low SOC content by long-term application of chemical fertilizer practice. Soil nitrogenase activity were increased with application of organic manure and

crop residue practices in ~~agree~~ with the previous studies [6, 27].

Effects of fertilizer regime on community structure of *cbbL*R and *nifH* gene

In the previous studies, the results indicated that the differences of soil physical and chemical properties can affect the community structure of nitrogen fixing microorganisms [28]. In this study, we found that the nitrogen-fixing bacteria community ~~were~~ more similar for different fertilizer treatments that were closer together. Different soil ecological environmental parameters ~~affect~~ the activity of soil autotrophic azotobacter and nitrogenase in particular. One of the key differences among the soil samples was the fertilizer treatments. The results indicated that fertilizer management may have been the strongest influence on the portion of the nitrogen-fixing bacteria community. Long-term application of crop residue and organic manure management (RF and OM treatments) had a greater diversity in the *cbbL*R and *nifH* genes, while application of chemical ~~fertilizer practice~~ (MF treatment) had a lower diversity in the *cbbL*R and *nifH* genes. Limmer and Drake (1996) [29] found that the levels of C and N influenced the activity and distribution of nitrogen fixation bacteria. In our previous study, MF and OM treatments had the highest concentrations of C and N [15], as well as the highest number of clones and diversity of *cbbL*R and *nifH* genes. In contrast, MF and CK treatments had the lowest C and N levels, and had smallest number of clones and diversity of *cbbL*R and *nifH* genes, respectively. Thus, the C and N content in paddy field may be the key factor~~s~~ influencing the nitrogen-fixing bacterial community.

There have been a large number of papers on the molecular diversity and phylogenetic analysis of *cbbL*R and *nifH* in different plant communities or geochemical environments using PCR-based analysis methods, giving rise to a large number of unidentified *cbbL*R and *nifH* clone sequences. In this study, the results showed that uncultured nitrogen fixing bacteria were found in the soil sample, and the other corresponding microorganisms with the highest homology belonged to *Proteobacteria*, *Betaproteobacteria*, *Pseudacidovorax*, *Azospira* and *Ideonella*, respectively. The results showed that the genetic diversity of azotobacter ~~were rich~~ in the paddy soil~~.~~ Jia et al. (2020) [8] ~~result~~ also proved that it is suitable for biological nitrogen fixation in rhizosphere soil of paddy field. In this study, some unknown *cbbL*R and *nifH* clone sequences were also found. The phylogenetic tree revealed some important patterns from the new nitrogen-fixing bacteria. (i) The *cbbL*R and *nifH* tree did not indicate~~d~~ that there was a clear relation ~~to~~ the different fertilizer treatments ~~sampling soils~~; however, there were clearly different dominant nitrogen fixing bacteria

populations at fertilizer treatments, which suggested that soil environmental parameters might be influencing the diversity and distribution of these microbial populations. (ii) The majority of the sequenced clones were not closely related to any known cultivated nitrogen-fixing bacteria. Fifty-five percent of the sequenced clones exhibited less than 72% nucleotide acid identity with known nitrogen fixation bacteria in the database, suggesting that they are unique and may represent novel sequences of nitrogen fixing community, and that most members of *cbbL*R and *nifH*-containing bacteria in the community might be unculturable. Provided that the clone libraries represent the in situ microbial community structure at the functional cluster level, the novel clusters of bacteria appear to be abundant in the study area. Real-time PCR and other molecular methods will be needed to estimate or quantify these novel clusters in order to verify their abundance in the soil. In order to understand their functionality, further cultivation strategies will also be needed to recover organisms with novel sequences.

This study first discussed the communities and structure of nitrogen fixing bacteria with different fertilizer regime under double-cropping rice field in southern China. As well as contributing information on their genetic diversity and providing some clone sequences of nitrogen fixation bacteria in double-cropping rice areas, we also present a view of the fertilizer management factors that affect this important group of bacteria. The effects of different soil environmental factors on bacteria communities associated with long-term changes need to be investigated in order to better understand the linkage between the functional processes and microbial community structures involved in nitrogen cycling.

Conclusion

In the present study, the results showed that number of cultivable nitrogen fixing microorganisms was significantly increased by long-term application of fertilizer treatments. The highest number of nitrogen fixing microorganisms with RF and OM treatments, followed by MF treatment. Meanwhile, this study indicated that diversity index and richness of *cbbL*R gene and *nifH* gene were increased under application of crop residue and organic manure condition. Soil nitrogenase activities were significantly enhanced under long-term application of organic manure and crop residue condition, but application of chemical fertilizer practice reduces soil nitrogenase activities. There is an obvious difference in characteristic of nitrogen fixing bacteria community

between application of inorganic fertilizer and organic manure treatments. Therefore, ~~it is a benefit practice to improve~~ soil nitrogen level in a double-cropping paddy field by application of organic manure and crop residue ~~practice~~. However, future studies ~~were~~ needed to investigate how changes of nitrogen fixing bacteria community under different ~~fertilizer practice~~ influence ~~on~~ ecological functions of rhizosphere microorganism.

Acknowledgements

This study was supported by National Natural Science Foundation of China (31872851), Innovative Research Groups of the Natural Science Foundation of Hunan Province (2019JJ10003).

References

1. Wang S, Pablo GP, Ye J, Huang DF. (2012) Abundance and diversity of nitrogen-fixing bacteria in rhizosphere and bulk paddy soil under different duration of organic management. *World J. Microbiol. Biotechnol*. 28: 493–503.
2. Greenland DJ. (1998) The Sustainability of Rice Farming. CAB International Publication in Association with the International Rice Research Institute, London, 110–113.
3. Lalucat J, Bennasar A, Bosch R, García-Valdés E, Palleroni NJ. (2006) Biology of Pseudomonas stutzeri. *Microbiol. Mol. Biol. Rev.* 70: 510–547.
4. Dos Santos PC, Dean DR. (2011) Co-ordination and fine-tuning of nitrogen fixation in Azotobacter vinelandii. *Mol. Microbiol.* 79: 1132–1135.
5. Teng QH, Sun B, Fu XR, Li SP, Cui ZL, Cao H. (2009) Analysis of nifH gene diversity in red soil amended with manure in Jiangxi, South China. *J. Microbiol*. 47: 135–141.
6. Tang Y, Zhang M, Chen A, Zhang W, Wei W, Sheng R. (2017) Impact of fertilization regimes on diazotroph community compositions and N_2-fixation activity in paddy soil. *Agric. Ecosyst. Environ.* 247: 1–8.
7. Kumar U. (2017) Diazotrophic microbes in rice: a boon to save nitrogen fertilizers. *Microbiol* 6: 1–3.
8. Jia R, Wang K, Li L, Qu Z, Shen WS, Qu D. (2020) Abundance and community succession of nitrogen-fixing bacteria in ferrihydrite enriched cultures of paddy soils is closely related to

Fe(III)-reduction. *Sci. Total Environ.*, 720: 137633.

9. Zehr JP, Mellon MT, Zani S. (1998) New nitrogen-fixing microorganisms detected in oligotrophic oceans by amplification of notrogenase (nifH) genes. *Appl Environ Microbiol* 64: 3444–3450.

10. Piceno YM, Lovell CR. (2000) Stability in natural bacterial communities. I. Nutrient addition effects on rhizosphere diazotrop assemblage composition. *Microb Ecol* 39: 32–40.,

11. Widmer F, Shaffer BT, Porteous LA, Seidler RJ. (1999) Analysis of nifH gene pool complexity in soil and litter at a Douglas fir forest site in the Oregon Cascade mountain range. *Appl Eviron Microbiol* 65: 374–380.

12. Shaffer BT,Wildmer F, Porteous LA, Seidler RJ. (2000) Temporal and spatial distribution of the nifH gene of N_2-fixing bacteria in forests and clearcuts in western Oregon. *Microb Ecol* 39: 12–21.

13. Rosch C, Mergel A, Bothe H. (2002) Biodiversity of denitrifying and dinitrogen-fixing bacteria in an acid forest soil. *Appl Environ Microbiol* 68: 3818–329.

14. Yang XY, Ren WD, Sun BH, Zhang SL. (2012) Effects of contrasting soil management regimes on total and labile soil organic carbon fractions in a loess soil in China. *Geoderma* 177–178: 49–56

15. Tang HM, Li C, Xiao XP, Pan XC, Cheng KK, Shi LH, et al. (2020) Effects of long-term fertiliser regime on soil organic carbon and its labile fractions under double cropping rice system of southern China. *Acta Agr. Scand B-S P.*, 70: 409-418.

16. Tang HM, Xiao XP, Tang WG, Li C, Wang K, Li WY, et al. (2018) Long-term effects of NPK fertilizers and organic manures on soil organic carbon and carbon management index under a double-cropping rice system in Southern China. *Commun. Soil Sci. Plan*, 49: 1976–1989.

17. Li ZG, Luo YM, Teng Y. (2008) Method of soil and environmental microorganisms. Beijing: Science Press, 97-99.

18. Schwinghamer EA, Dudman WF, Cannon FC. (1980) In: Bergersen, F.J. (Ed.), Methods for Evaluating Biological Nitrogen Fixation. John Wiley and Sons, UK, Chichester, 111–138.

19. Poly F, Monrozier LJ, Bally R. (2001) Improvement in the RFLP procedure for studying the diversity of nifH genes in communities of nitrogen fixers in soil. *Res. Microbiol.* 152: 95–103.

20. Muyzer G, De-Waal EC, Uitterlinden AG. (1993) Profiling of complex microbial populations

by denaturing gradient gel electrophoresis analysis of polymerase chain reaction-amplified genes coding for 16S rRNA. *Appl. Environ. Microbiol.* 59: 695–700.

21. Fagen JR, Giongo A, Brown CT, Davis-Richardson A, Gano KA, Triplett EW. (2012) Characterization of the relative abundance of the citrus pathogen Ca. Liberibacter asiaticus in the microbiome of its insect 525vecor, Diaphorina citri, using high throughput 16S rRNA sequencing. *Open Microbiol J*. 6: 29–33.

22. Luo ZX, Chen Z, Qiu ZZ, Li YC, Laing GD, Liu A, (2015) Gold and silver nanoparticle effects on ammonia-oxidizing bacteria cultures under ammoxidation. *Chemosphere* 120: 737–742.

23. SAS. (2008) SAS Software of the SAS System for Windows. SAS Institute Inc, Cary, NC, USA.

24. Yuan HZ, Ge TD, Wu XH, Liu SL, Tong CL, Qin HL, et al. (2012)Long-term field fertilization alters the diversity of autotrophic bacteria based on the ribulose 1,5-hiphosphate carboxylase/oxygenase (RubisCO) large subunit genes in paddy soil. *Appl Microbiol Biot*, 95: 1061-1071.

25. Zhou P, Pan GX, Spaccini R. (2010) Molecular changes in patticulate organic matter in a typical Chinese paddy soil under differen long-term fertilizer treatments. *Eur J Soil Sci*., 61: 231-242.

26. Das S, Bhattacharyya P, Adhya TK. (2011) Impact of elevated CO_2, flooding, and temperature interaction on heterotrophic nitrogen fixation in tropical rice soils. *Biol. Fertil.Soils* 47: 25–30.

27. Tanaka H, Kyaw KM, Toyota K, Motobayashi T. (2006) Influence of application of rice straw, farmyard manure, and municipal biowastes on nitrogen fixation, soil microbial biomass N, and mineral N in a mode paddy microcosm. *Biol. Fertil.Soils*, 4: 501–505.

28. Baumann K, Marschner P, Smernik RJ, Baldock JA. (2009) Residue chemistry and microbial community structure during decomposition of eucalypt, wheat and vetch residues. *Soil Biol Biochem,* 41: 1966-1975.

29. Limmer C, Drake HL. (1996) Non-symbiotic N_2-fixation in acidic and pH-neutral forest soil: aerobic and anaerobic differentials. *Soil Biol Biochem*, 28: 177–183.

Figure legend

Figure 1 Effects of different long-term fertilizer treatments on soil autotrophic azotobacter (a) and soil nitrogenase activities (b)

MF: chemical fertilizer alone; RF: rice straw residue and chemical fertilizer; OM: 30% organic manure and 70% chemical fertilizer; CK: without fertilizer input as control.

Error bars represent standard error of mean. Different letters are significantly different at $p<0.05$ level.

The same as below.

Figure 2 Similarity dendrograms (UPGMA, Dice coefficient of similarity) analysis of *cbbL*R gene (a) and *nifH* gene (b) under different fertilizer treatments

Figure 3 Phylogenetic tree of *cbbL*R gene of samples using all OTUs identified under different fertilizer treatments

Figure 4 Phylogenetic tree of *nifH* gene of samples using all OTUs identified under different fertilizer treatments

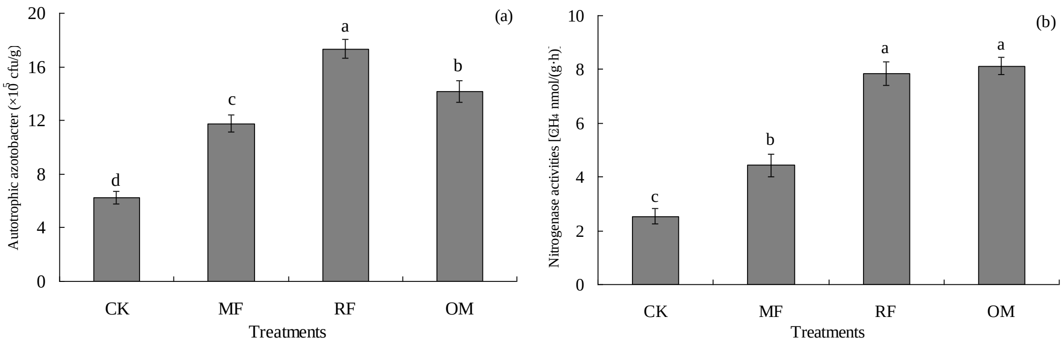

Figure 1 Effects of different long-term fertilizer treatments on soil autotrophic azotobacter (a) and soil nitrogenase activities (b)

MF: chemical fertilizer alone; RF: rice straw residue and chemical fertilizer; OM: 30% organic manure and 70% chemical fertilizer; CK: without fertilizer input as control.

Error bars represent standard error of mean. Different letters are significantly different at $p<0.05$ level.

The same as below.

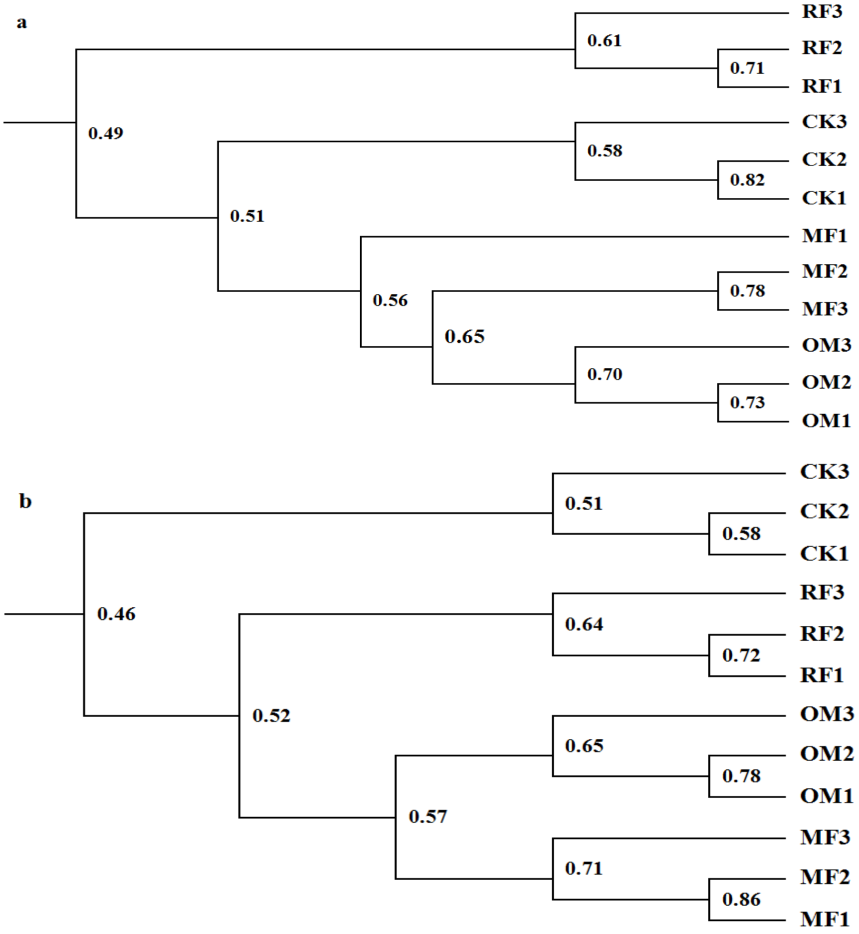

Figure 2 Similarity dendrograms (UPGMA, Dice coefficient of similarity) analysis of *cbbL*R gene (a) and *nifH* gene (b) under different fertilizer treatments

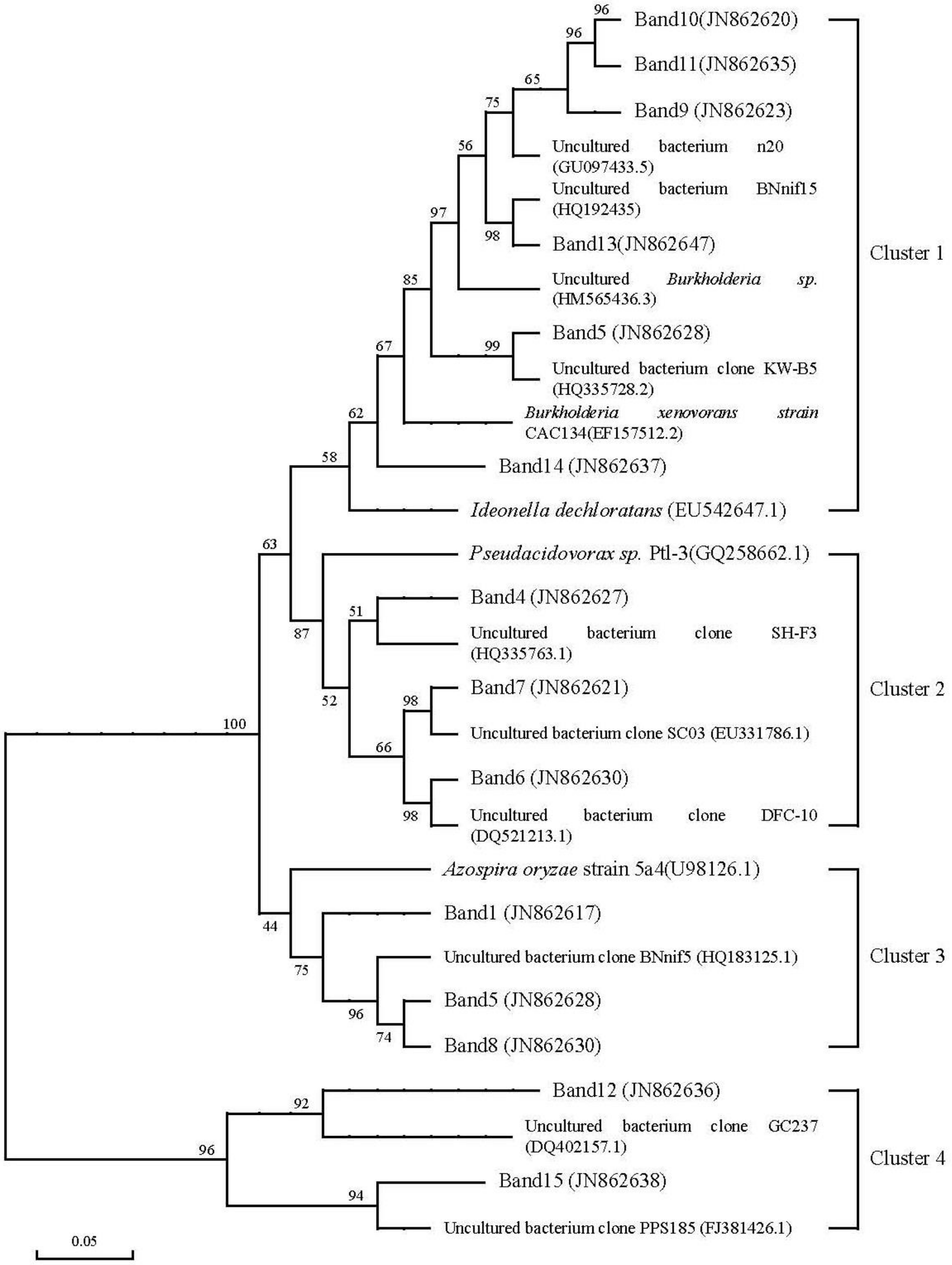

Figure 3 Phylogenetic tree of *cbbL*R gene of samples using all OTUs identified under different fertilizer treatments

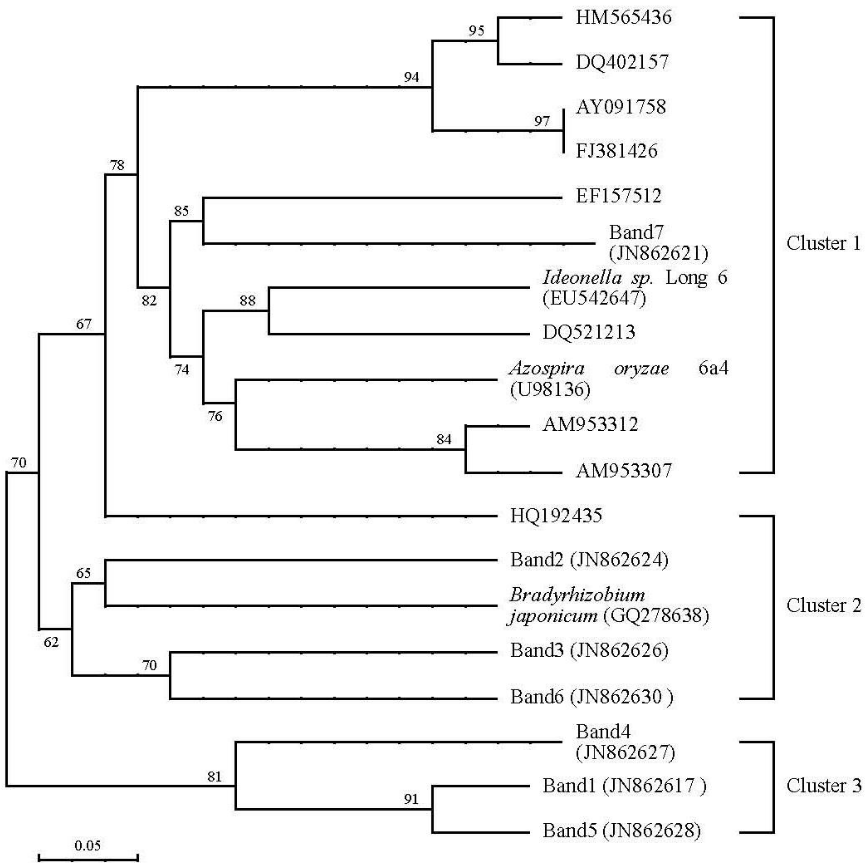

Figure 4 Phylogenetic tree of *nifH* gene of samples using all OTUs identified under different fertilizer treatments
